# ERP Modulation during Observation of Abstract Paintings by Franz Kline

**DOI:** 10.1371/journal.pone.0075241

**Published:** 2013-10-09

**Authors:** Beatrice Sbriscia-Fioretti, Cristina Berchio, David Freedberg, Vittorio Gallese, Maria Alessandra Umiltà

**Affiliations:** 1 Department of Neuroscience, University of Parma, Parma, Italy; 2 Department of Fundamental Neuroscience, University of Geneva, Geneve, Switzerland; 3 Department Of Art History and Archaeology, Columbia University, New York City, New York, United States of America; University of Rome, Italy

## Abstract

The aim of this study was to test the involvement of sensorimotor cortical circuits during the beholding of the static consequences of hand gestures devoid of any meaning.In order to verify this hypothesis we performed an EEG experiment presenting to participants images of abstract works of art with marked traces of brushstrokes. The EEG data were analyzed by using Event Related Potentials (ERPs). We aimed to demonstrate a direct involvement of sensorimotor cortical circuits during the beholding of these selected works of abstract art. The stimuli consisted of three different abstract black and white paintings by Franz Kline. Results verified our experimental hypothesis showing the activation of premotor and motor cortical areas during stimuli observation. In addition, abstract works of art observation elicited the activation of reward-related orbitofrontal areas, and cognitive categorization-related prefrontal areas. The cortical sensorimotor activation is a fundamental neurophysiological demonstration of the direct involvement of the cortical motor system in perception of static meaningless images belonging to abstract art. These results support the role of embodied simulation of artist’s gestures in the perception of works of art.

## Introduction

Early neuroscientific interest in the relationship between art and brain began to emerge in works by Changeux and Zeki [1], [2] in the 1990s. The first research questions generally concerned the visual processes underlying aesthetic experience [3], [4], [5]. Which are the visual features leading a particular experience to be perceived as aesthetic? Such studies emphasized the distinctive coincidence between the cortical organization of the visual nervous system and the visual attributes that artists use to express their art. The assumption was that the implicit and unconscious goals of an artist coincided to some degree with the basic functioning of vision. In this sense, abstract artworks could be taken as offering effective examples of this position, given that they could be said to extract primitive perceptual principles from reality [6] – with regard to color for example, in the case of Matisse’s works, or motion, in the case of Calder’s. Other researches investigated the neural basis of aesthetic experience from the point of view not only of early perceptual but also of higher order processes accompanying aesthetic experience (including aesthetic judgment) [7], [8] and reward mechanisms [9], [10].

Although the activation of the visual cortex of the beholder is a crucial aspect of visual art perception, we believe that the “power of images” [11] is neither exclusively referable just to a cortical visual process nor to high order cognitive processes. In our view, automatic and pre-reflexive activations, involving emotional and sensorimotor cortical circuits, should be a distinctive aspect of responses to visual art [12], [13], [14]. The perception of visual stimuli does not only involve visual cortical areas. Indeed, many studies demonstrated that visual perception, in most cases, required the activation of sensorimotor circuits [15]. In this sense perception and action are not separate domains implemented in different anatomical and functional circuits, as maintained by the traditional view represented by the “sandwich” model of cognition [16]. In other words, we do not first perceive and then act, action itself contributes to perception.

The existence of visual and motor responses embedded in the same neuron was demonstrated with the discovery of mirror neurons in the ventral premotor cortex of macaque monkey [17]. Mirror neurons discharge both when a certain action is executed and when a similar action is observed [18], [19], [20]. Mirror neurons have been subsequently recorded also in the Inferior Parietal Lobule (IPL) of the macaque monkey [21], [22], [23]. Neuroimaging, Transcranial magnetic Stimulation (TMS), and single neuron recordings experiments, have demonstrated also in humans the existence of a mirror mechanism, matching action observation with action execution, similar to that previously discovered in the monkey [24], [25]. Further studies showed that a similar mirror mechanism is likely involved when perceiving the emotions and sensations of others [25], [26]. This led to the formulation of the theory of embodied simulation [27], [28], [29]. Embodied simulation provides a new empirically based notion of intersubjectivity, viewed first and foremost as intercorporeality. By means of embodied simulation we can map others’ actions by re-using our own motor representations, as well as others’ emotions and sensations by re-using our own viscero-motor and somatosensory representations. The relevance of embodied simulation for the perception of works of art was recently proposed [12]. These authors proposed that the relationship established between the beholder and the artwork is based on the activation of sensorimotor brain circuits, allowing the observer to establish an embodied relation with the content of the observed artwork. The tight relation between action and perception implies the contribution of sensorimotor circuits during perceptive processes activated by the observation of visual artworks. Zeki and Ramachandran hypothesized the artist’s unconscious ability to emphasize visual elements capable of activating “the visual brain” of the observer; our proposal is to postulate also the artist’s ability to activate viewers’ sensorimotor circuits.

Activation of cortical motor areas (identified as µ suppression) can be simplyinduced by the observation of static pictures of grasping [30]. Furthermore, a recent ERP study demonstrated a direct relation between the observation of static images and the activation of the cortical motor system [31]. These authors showed that, during presentation of pictures representing human actions with different degrees of dynamism, there was a greater motor cortical activation for observation of pictures representing more dynamic actions than for observation of pictures representing less dynamic actions. The source localization analysis indicated that the observation of more dynamic images was accompanied by the activation of a series of cortical regions belonging to the motion and action representation systems, namely: V5/MT (Middle temporal visual area), EBA (Extrastriate Body Area), STS (Superior Temporal Sulcus), premotor and motor areas. In particular, the activation, among others of premotor and motor areas emerged. Although these studies did not directly address the question of the neural mechanisms at the basis of perception of visual art images, these results suggest that, even in the absence of explicit motion, the implicit movement represented in a static image is sufficient to activate the cortical motor circuits that are recruited during the actual execution of the observed movement. Other studies showed stronger cortical motor activation during the observation of handwritten letters compared with printed letters [32], [33], [34]. These studies demonstrated that this activation corresponds to a covert motor simulation of movements leading to a static graphic outcome and that it is part of the perceptual process activated by the observation of hand written letters. These results suggest the presence of a motor simulation mechanism not only of static representations of actions [31], but also a motor simulation mechanism allowing an ‘a posteriori’ reconstruction of the agent’s action executed to achieve the graphic consequence itself [32], [33], [34]. Two recent EEG studies directly linked the observation of static images with the activation of mirror mechanism showing that the observation of static images selected from Rorscharch test evoked the activation of the sensory motor cortex [35], [36]. The authors claimed that the mere observation of Rorcharch cards is able to evoke the activation of the mirror mechanism.

In line with these studies, we hypothesized that also the observation of brush strokes, as visible traces of goal-directed movements, are capable of activating the cortical representation of the same hand gesture in the observers’ brain. In order to verify this hypothesis we performed an EEG experiment presenting to participants images of abstract works of art characterized by the presence of marked traces of brushstrokes. We aimed to offer a closer assessment of the involvement of sensorimotor cortical circuits during the beholding of the static consequences of hand gestures devoid of any meaning, thus different from those employed by the majority of previous studies, which employed handwritten letters. The stimuli consisted of three different black and white paintings by Franz Kline, an artist belonging to the Abstract Expressionism movement of the New York School of the 1940s, 1950s and 1960s (together with, among others, Jackson Pollock and Willem de Kooning). The works selected as stimuli for the experiment are characterized by a small number of brushstrokes, thus allowing for an easier construction of three reliable control images. The EEG data were analyzed by using Event Related Potentials (ERPs) as a means for assessing the difference between the observation of three Paintings and the observation of three Modified stimuli while by means of source localization, we individuated the cortical areas differently activated under these two experimental conditions.

## Method

### Participants

Twenty-one healthy adult volunteers (11 females, 10 males) whose age ranged between 21 and 34 years (mean age 28; sd +/−4.07) participated in the experiment. All participants were right handed, as tested by Edinburgh Handedness Inventory [37]. Participants had normal or correct to normal vision and none had previous psychiatric or neurological history. Before the experiment, they received oral information about the experiment procedures and gave their written informed consent to the experiment. Participants were recruited by public announcement. All participants had pursued graduate studies, none had specific experience in painting or in art theory, none knew the experimental goals. The experiment was approved by the Local Ethical Committee. The experiment was approved by the Local Ethical Committee (Comitato Etico Unico per la Provincia di Parma, Italy). +39.0521.703013-702449- F. +39.0521.704702.

### Stimuli

**Figure 1:**
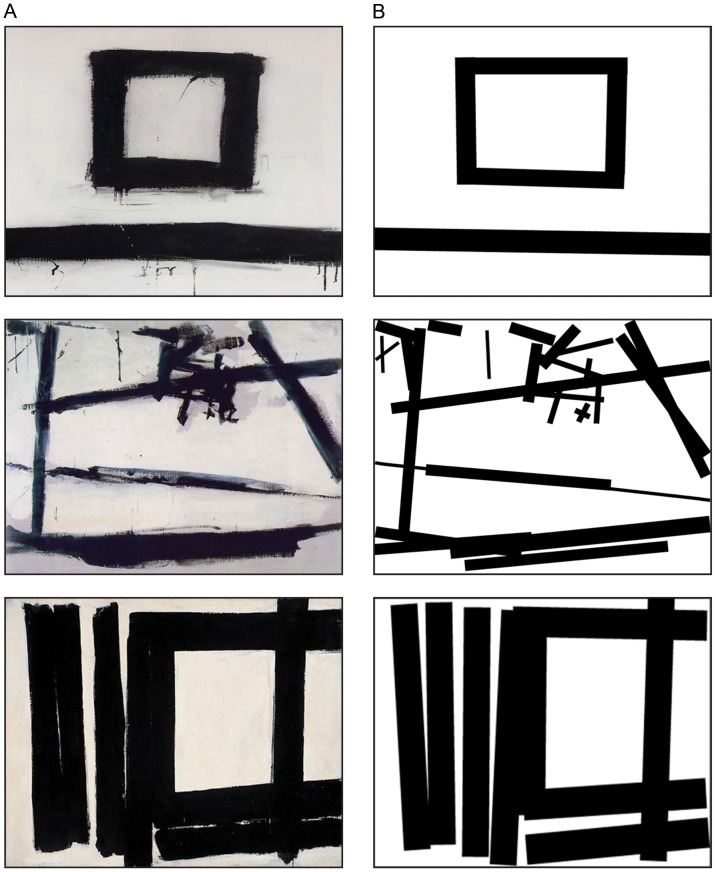
Stimuli used in the study. (A) Painting stimuli: from up to bottom: 1953 Suspended; 1954 Painting number 2 and 1952 Painting Number 7 (B) Modified stimuli created removing the dynamic components of the three original paintings.

We used two different categories of stimuli: Paintings and Modified stimuli (see Figure 1, A and B). Paintings stimuli (see Figure 1A) consisted of digital images of three abstract black and white paintings by Franz Kline: 1953 *Suspended*, 1954 *Painting Number 2*, and 1952 *Painting Number 7*. These images were downloaded from open source web sites. Modified stimuli were altered versions of the three Paintings stimuli, built using Adobe Photoshop® software. In the Modified stimuli (see Figure 1B) the dynamic components of the original Paintings were removed by replacing them with graphic dense black stripes of a similar thickness and length, keeping the same graphic pattern and simmetry as the actual paintings stimuli. The Modified stimuli were designed with the intent of removing the visible consequences of the artist’s gesture (such as drips of paint, blurred contours, differences in pressure on the brush, and so on).

In order to exclude the possibility that the two categories of stimuli could elicit a different tendency to move the eyes, we counted the number of eye blinks and eye movements of each participant in both experimental conditions. Mean eye blinks: 3.94 (SE +/−1.3) for Paintings and 4.0 (SE +/−1.3) for Modified stimuli. Mean eye movements: 3.26 (SE +/−1.1) for Paintings and 3.52 (SE +/−1.6) for Modified stimuli. T-test applied in order to compare eye blinks and eye movements in the two conditions did not produce a statistically significant result (both Ps>0.7). So we can exclude that participants used different patterns of saccadic movements to explore the two categories of presented images.

### Procedure and Experimental Paradigm

Participants were seated in a comfortable chair, placed in an isolated, dimly lit room, in front of a 17-inch computer monitor used for stimuli presentation and located at a distance of 70 cm from participant’s body. In order to minimize movements and blink artifacts during the recording session, participants were asked to keep their forearms resting on a table in front of them and to avoid any eye or body movement. Participants were instructed to gaze at the center of the screen. The experiment included two different sessions: an EEG recording session, and at the end of it, a behavioral rating session. The duration of the entire experiment was around 20 minutes. Each EEG trial started with the presentation of a black screen of variable duration from 4,5 to 5 seconds (rest), followed by a fixation cross (450 ms to 550 ms) and by stimuli presentation (1 s). In 50% of the trials (attentive trials) the disappearance of the stimulus was followed by the presentation of a green or a red dot. Participants were asked to give a verbal response as to the color of each dot. These trials were presented in order to keep participants attention, and the verbal responses time periods were not used for any subsequent analyses. The six stimuli (3 Paintings and 3 Modified stimuli) were randomly presented 15 times each. In total each participant was presented with 90 stimuli.

After completing the EEG recording session, participants were asked to fill out a questionnaire related to the images just observed. Watching again the six images in random order, participants were asked to assess four different parameters for each image: 1) Familiarity (“If and how are you familiar with this image”, score from 0 to +10) 2) Aesthetic appraisal (“How much do you like this image?” score from −10 to +10); 3) Amount of movement (“How much movement do you perceive in this image”, score from 0 to +10) 4) Artistic nature of the perceived images (“Is the image a real artwork?”, score 1 if the answer was “yes” and 0 if the answer was “no”).

#### EEG recording

EEG data were acquired by a 128-channel Sensor Net (Electrical Geodesic, Eugene, USA) and recorded within standard EGI package Net Station 4.3.1. EEG was sampled at 250 Hz and band-pass filtered at 0.3–100 Hz. Electrodes impedance was kept below 50 KΩ. Raw EEG data were recorded with the vertex (Cz) as the online reference and re-referenced off-line to a linked-mastoid reference. Stimuli were delivered with E-prime 2.0. E-prime sent to Net Station all different markers related to the beginning of each event (black screen, fixation cross, experimental stimulus presentation, attentive stimulus presentation) and labeled the experimental conditions and events. The experimenter, seated in front of the acquisition computer monitor, checked participants movements through a video camera synchronized with the EEG traces. When participants moved, the trial was automatically excluded from further analysis.

#### EEG data analysis

Data were analyzed using the Net Station software. Data were filtered offline with a band-pass filter 1–30 and re-referenced to a linked-mastoid reference using as reference the average signal registered from electrodes 57 and 100. This reference is considered suitable to minimize the alteration of the signal coming from the central areas of the brain [38]. The EEG signal was segmented into 1,200 ms epochs (single trial) starting 200 ms before the stimulus appearance and ending 1000 ms after it. Each segment included 200 ms corresponding to the fixation cross (considered as the baseline) and the entire duration of the stimulus presentation period (1000 ms). Trials with eye blinks, eye movements and muscular artifacts were identified and rejected on the basis of the Net Station artifacts detection tool. In addition, two experimenters made a double blind careful visual inspection of all segments to detect residual artifacts contamination. Two participants, having a number of artifact-free trials that was less than 50%, were discarded. The average number of artifacts-free trials kept for statistical analyses was 32.7 (SE ±1.6; 72,6%) for the Paintings and 32.9 (SE ±1.9, 73,1%) for the Modified stimuli. T-Test was performed to compare the number of artifact-free trials between the two conditions and no statistically significant difference was found (p>0.5).

**Figure 2:**
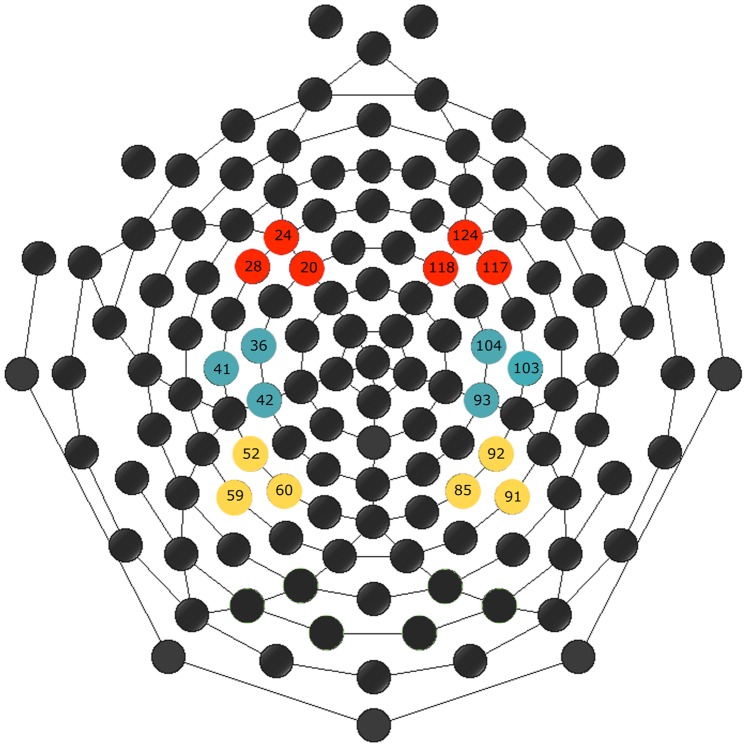
Clusters of electrodes selected for the analysis of ERP component. Six different clusters, of three electrodes each, have been selected: 2 frontal (red), 2 central (blue), and 2 parietal (yellow).

For each participant, EEG data from 200 ms before stimulus presentation onset (baseline) through 1000 ms after stimulus onset were averaged off-line. The grand average was obtained averaging the single averages of all participants. In order to select the temporal windows to be used for ERP analyses, a T-test was conducted between the two conditions on the signal amplitude of each participant. T-test was conducted for the entire duration of the temporal segment and on all 128 electrodes (Net Station software tool, p</ = 0,01). The selected temporal window (260–328 ms after stimulus presentation) was used for the ERPs peak latency and mean amplitude analyses. Different clusters of electrodes were used for statistical extraction: three electrodes around F3–F4 (24-28-20; 124-118-117), three electrodes around C3–C4 (36-41-42; 104-103-93) and three electrodes around P3–P4 (52-59-60; 85-91-92, see Figure 2). ERPs mean amplitude and latency were averaged between electrodes of each cluster.

In order to assess ERP differences between conditions in the mean amplitude and peak latency, we performed two separated 3X2X2 repeated measures ANOVAs on the negative component identified in the selected temporal windows (260–328 ms after the stimulus presentation). Both the ANOVAs had Cortical Region (Frontal, Central and Parietal), Hemisphere (Left and Right) and Condition (Paintings vs. Modified stimuli) as within subjects factors. Post-hoc tests (Newman-Keuls, test p< = 0,05) were applied on all significant main factors and interactions.

#### ERPs source localization

Standardized Low-Resolution Brain Electromagnetic Tomography (s-LORETA, 39) software (downloaded from academic open source web site http://www.uzh.ch/keyinst/loreta.htm) was used for source localization analysis. The cortical three-dimensional distribution of current density was calculated in order to localize the optimal neuroanatomical generators of the statistically significant negative component, in the selected time window (see below).

The sLORETA allows a tomography with unbiased localization [39], [40], [41], although with low spatial resolution. The 3D brain compartment of the model is restricted to the cortical gray matter/hippocampus of a realistic head model [42], as determined by the probabilistic Talairach atlas [43], using the MNI152 adult template. On this template the sLORETA software computes the electrodes position taken from Jurcak 2007 [44] and Oostenveld and Praamstra 2011 [45]. The brain compartment includes 6239 voxel, each one corresponding to an equivalent current dipole as theorized by distributed models. Analyses were conducted on the averaged intra-subject differential signal of Paintings minus Modified stimuli. The current density resulted from the subtraction between conditions (Paintings - Modified stimuli) was computed for the 6239 brain matter voxels in the whole duration of the segment (1200 ms). From the obtained differential signal, two different time windows were extracted for the statistical analysis. The first time window (observation Paintings - Modified stimuli) was of 268–320 ms (52 ms equivalent to 13 time frames of 4 ms each) and encompassed the negative ERP component previously identified by the T-test conducted on the single averages of all participants between the two conditions (see above in 2.5 section). The second time window lasted from −64 to −12 ms before stimulus presentation, equivalent to 52 ms and 13 time frames. By means of a T-test we compared the current densities of the differential signal of these two time windows.

#### Questionnaire analysis

The scores of “Familiarity”, “Aesthetic appraisal”, “Amount of movement” and “Artistic nature” that participants gave to the 3 Original and 3 Modifed stimuli were averaged in order to have 2 averages for each of the four questionnaires for both experimental conditions. Four different T-tests were then applied on the resulted averages in order to compare the scores of each questionnaire for the Original and Modified stimuli.

## Results

### Time Window Detection

**Figure 3:**
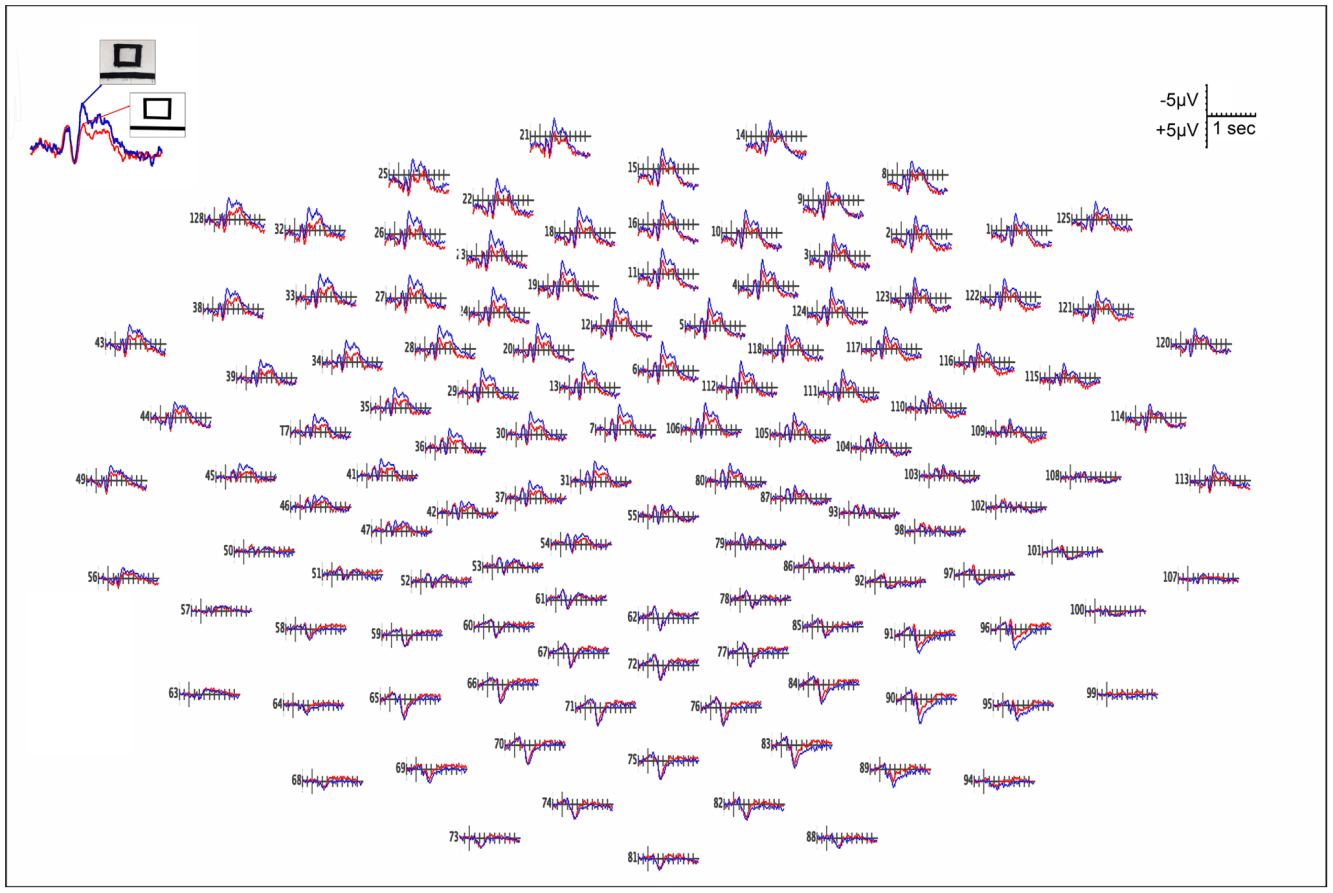
Grande-average ERP waveform (19 participants). The different colors indicate the 2 different Conditions: Paintings (blue line) and Modified stimuli (red line).

Figure 3 shows the grand average ERP waveform of the scalp as function of condition: Paintings (blue line) and Modified stimuli (red line). A negative fronto-central deflexion is evident and it is modulated between the two conditions, being much larger during the observation of Paintings than during the observation of Modified stimuli.

**Figure 4:**
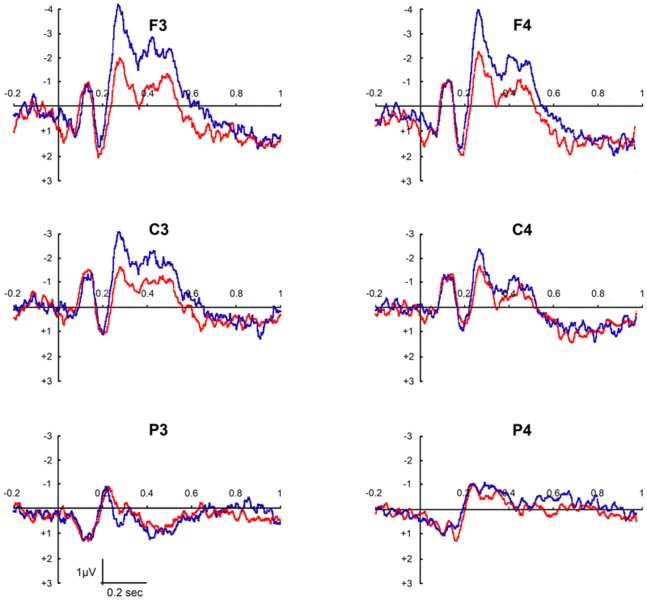
ERP waveforms recorded over frontal (F3 and F4), central (C3 and C4) and parietal (P3 and P4) sites. The different colors indicate the 2 different Conditions: Paintings (blue line) and Modified stimuli (red line). Paintings evidently have a greater effect especially on the signal recorded on left frontal and central sites.

Figure 4 shows the grand average ERP waveform recorded from Frontal (F3 and F4), Central (C3 and C4) and Parietal (P3 and P4) electrodes in both Conditions.

**Figure 5:**
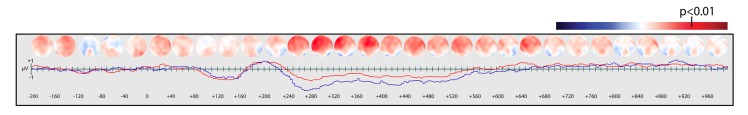
T-test applied on the two conditions (Paintings vs Modified stimuli). It was conducted on bins of 40(from −200 to 1000 ms). In the upper part of the figure the t values topomaps are shown. The lower part of the figure shows the modulation, in the two experimental Conditions (blue line = Paintings; red line = Modified stimuli), of grand averaged signal recorded from C3. 240 ms after stimulus presentation the two conditions significantly differ on the fronto-central electrodes.

In order to select an appropriate time window for further statistical analyses, a T-test (Paintings vs. Modified, p< = 0,01) has been applied on the entire segment temporal length (1200 ms). T-test comparison resulted in the occurrence of a fronto-central statistically significant difference (p<0.01) in the amplitude signal, starting 240 ms after stimulus appearance (Figure 5) and ending 400 ms after it. In order to include the peak of this negative ERP component, we took a time window from 260 to 328 ms after stimulus presentation. This negative ERP component was used to conduct two repeated measures ANOVAs, one on the ERP mean amplitude and one on the peak latency.

### ERP Negative Component Analysis

**Figure 6:**
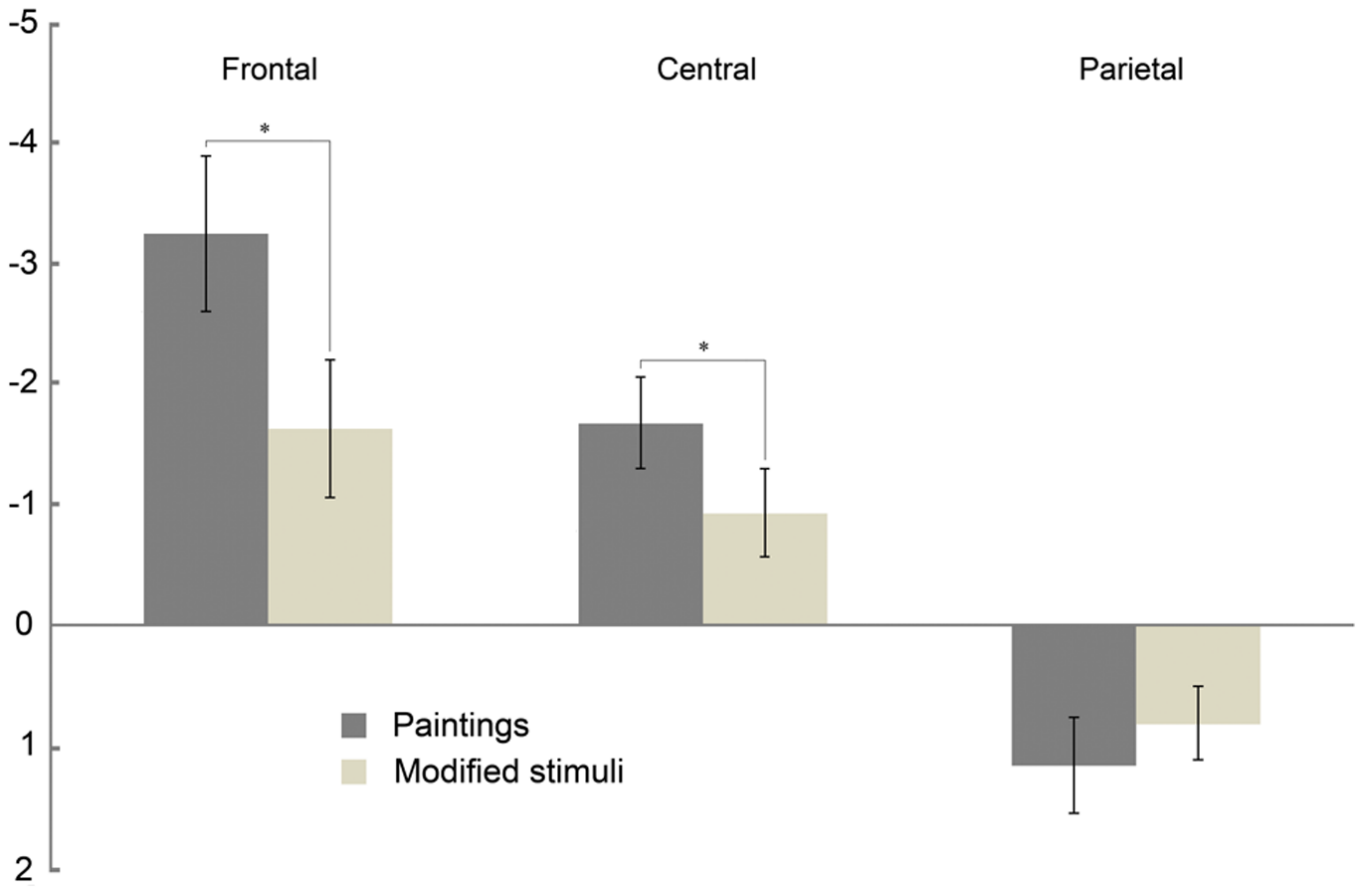
Modulation of the negative ERP component during observation of Paintings and Modified stimuli. Bars represent significant RegionXCondition interaction resulted from the ANOVA performed on the mean amplitude (µV). Paintings (dark gray) and Modified stimuli (light gray). In the frontal and central electrodes the two conditions are significantly different (*).

Results of the repeated measures ANOVA performed on the amplitude of ERP negative component showed a significant main effect of Region (F(2, 36) = 23,2 p<0.001) and Condition (F(1, 18) = 5,2 p<0.05). The same analysis revealed two significant interactions: RegionXCondition (F(2, 36) = 24,1 p<0.001) and HemisphereXCondition (F(1, 18) = 8,3 p<0.01). Post-hoc applied on the main effect of Region showed significantly higher amplitude of the negative ERP in the Frontal region, intermediate amplitude in the Central region, and finally, the lowest amplitude value in the Parietal Region (all p_s_ <0,05). More interestingly, the post-hoc comparisons, applied on Condition main effect, highlighted significantly higher negative ERP amplitude during the observation of Paintings than during the observation of Modified stimuli (p<0.05). The investigation of the RegionXCondition interaction (see Figure 6) showed a significant higher ERP amplitude when participants observed Paintings compared to the observation of Modified stimuli only in the Frontal (p<0.001) and Central Regions (p<0.005), but not in the Parietal electrodes (p>0.05). In addition, the ERP amplitude, evoked by the observation of Paintings, decreased moving from Frontal to Central and Parietal Regions (all p_s_ <0.001) as well as it occurred during the observation of Modified stimuli (all p_s_ <0.005).

The ANOVA performed on latency peak values of the negative ERP component resulted only in a significant main effect of Region (F(1, 36) = 10,5 p<0.001). Newman-Keuls post-hoc test applied on the significant main effect of Region showed a greater latency of the negative component in the Parietal region compared to the Frontal and the Central areas (all p_s_ p<0.001).

### Source Localization

Table 1 lists the cortical regions with significantly different (p<0.01) activations obtained comparing observation (observation of Painting Stimuli (PS) – observation of Modified Stimuli (MS)) with baseline (baseline PS-MS) time windows. The significant activations have been divided in four different clusters on the basis of their functional significance. The first cluster included the visual areas, the second incorporated areas belonging to the sensorimotor circuits, the third cluster included the left prefrontal areas, and finally, the fourth cluster encompassed the orbito-frontal regions.

**Table 1:** Significant activations (p<0.01) resulted from the sLORETA source analysis.

Region		x	y	Z	t value	k
VISUAL ACTIVATIONS						
Right Transverse Temporal Gyrus	Brodmann area 42	60	−10	10	6,61	3
Right Transverse Temporal Gyrus	Brodmann area 41	55	−15	10	6,50	1
Left Angular Gyrus	Brodmann area 39	−45	−75	35	5,83	1
Right Lingual Gyrus	Brodmann area 17	10	−90	0	5,77	1
Left Middle Temporal Gyrus	Brodmann area 21	−65	−50	−5	5,60	3
SENSORIMOTOR ACTIVATIONS						
Left Superior Parietal Lobule	Brodmann area 7	−25	−65	60	8,23	7
Right Inferior Parietal Lobule	Brodmann area 40	65	−25	30	7,02	4
Right Precentral Gyrus	Brodmann area 4	40	−25	65	6,99	6
Right Superior Temporal Gyrus	Brodmann area 22	60	−5	10	6,65	6
Right Precentral Gyrus	Brodmann area 6	60	0	10	6,63	7
Right Precentral Gyrus	Brodmann area 43	55	−10	10	6,62	3
Left Inferior Frontal Gyrus	Brodmann area 47	−25	30	−5	5,90	6
PREFRONTAL ACTIVATIONS						
Left Superior Frontal Gyrus	Brodmann area 9	−20	55	35	7,35	4
Left Medial Frontal Gyrus	Brodmann area 25	−5	25	−20	6,38	6
Left Superior Frontal Gyrus	Brodmann area 8	−20	30	50	6,34	5
Left Middle Frontal Gyrus	Brodmann area 8	−25	30	50	6,29	2
Left Inferior Frontal Gyrus	Brodmann area 47	−25	30	−5	5,90	6
Left Superior Frontal Gyrus	Brodmann area 10	−25	55	30	5,87	2
ORBITOFRONTAL AND CINGULATE ACTIVATIONS						
Left Middle Frontal Gyrus	Brodmann area 11	−35	55	−10	7,03	4
Right Orbital Gyrus	Brodmann area 11	5	45	−20	6,44	6
Right Medial Frontal Gyrus	Brodmann area 11	5	40	−15	6,43	7
Right Anterior Cingulate	Brodmann area 32	5	40	−10	6,22	2
Left Subcallosal Gyrus	Brodmann area 11	−10	25	−15	6,03	1
Right Superior Frontal Gyrus	Brodmann area 11	10	55	−25	6,02	3
Right Inferior Frontal Gyru	Brodmann area 11	10	40	−20	5,98	1

Coordinates are given in MNI space. Coordinates of each cortical region represent the peak voxel. The number of significant voxel in a region is identified as k.

**Figure 7:**
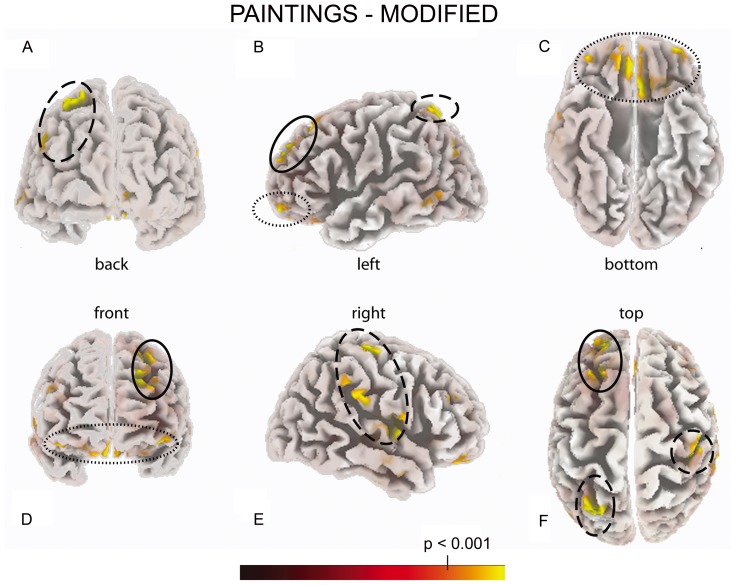
sLORETA inverse solution applied on the difference wave ‘Paintings – Modified Stimuli’. The neural generators resulted in the following cortical areas: sensorimotor (dashed lines), left prefrontal (continuous lines) and orbitofrontal (dotted lines), are evidenced.

The above mentioned ERP sources are visualized in Figure 7. The ERP sources that could be classified as part of sensorimotor circuits are statistically significant, and mainly localized on the right hemisphere (dashed lines). In particular the areas with a higher T values were: BA 40 (panel E), BA 4 (panels A, E and F), BA 22 (panel E) and BA 6 (panels A and E). On the left hemisphere one significant ERP sensorimotor source has been identified and it corresponded to BA 7 (panel B and F). The prefrontal cluster (continuous lines) was localized on the left hemisphere and was represented by Medial (BA 25), Superior (BA 8, BA 9 and BA 10) and Middle (BA 8) Frontal gyrus (panels B, D and E). The orbitofrontal cluster (dotted lines) was formed by bilateral activation of BA 11 and an additional source localized in the right BA 32 (panels B, C and D).

### Questionnaire Analysis

**Figure 8:**
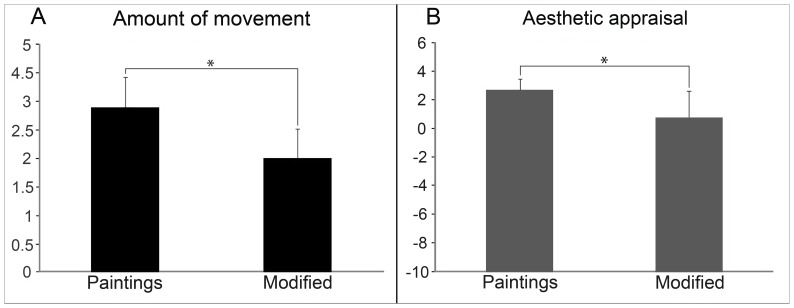
Results of the T-test conducted on the scores of the questionnaire. Scores given to the “Amount of movement” and to the “Aesthetic appraisal” resulted significantly higher for the Paintings than for the Modified stimuli (p_s_<0,01).

The results of the T-test applied on the averaged score that participants gave to the “Amount of movement” showed a significant higher score (p<0,01) for Paintings than Modified stimuli. A similar result was obtained with the averaged score that participants gave to the “Aesthetic appraisal”, being the Aesthetic appraisal significantly higher (p<0,001) for Paintings than Modified stimuli (see Figure 8). The averages of the Familiarity score participants gave to the 3 Original and 3 Modified stimuli were 1,90 (SE +/−0,56) and 1.00 (SE +/−0,41) indicating that they were poorly familiar with both types of stimuli. In addition, the T-test resulted not significant (p = 0,22), thus showing that participants were equally poorly familiar to Modified and Paintings images. The averages of the percentage of the Artistic nature score were 87,71 (SE +/−3.50) for the 3 Original and 33.33 (SE +/−1,75) for 3 Modified stimuli. T-test applied on these two averages resulted significant (p<0,001) indicating that Original stimuli were perceived as original art works while Modified stimuli were not.

## Discussion

The aim of this study was to investigate the cortical motor responses to the observation of static works of abstract art by Franz Kline. We sought to establish whether the implicit movement existing in the images shown, as brushstroke traces, was sufficient to activate the cortical motor circuits recruited by the artist during the actual execution of the observed brushstrokes.

We recorded EEG signals during the passive observation of digital images of three different artworks by Franz Kline (Paintings Stimuli) and three control images (Modified stimuli). The control images were designed in order to reduce the dynamic components of the original Paintings, while keeping the same graphic pattern. What should be emphasized here is that the experimenters always named all stimuli as “images” without advising participants of their artistic relevance.

The results of the ERP mean amplitude analyses showed that observation of Paintings produced a clear and significantly greater cortical activation than observation of modified stimuli. This difference lays in a negative deflection occurring from 260 to 328 ms after stimulus presentation at frontal and central scalp sites. Such negativity can be compared with a similar component present in an ERP study focused on the comparison between action observation and execution [46], demonstrating a centrolateral and parietal negativity during the observation of finger movements. These authors proposed that this negative ERP, accompanying movement observation, might correspond to the negative MRPs (Motor Related Potentials) found during motor execution [47], [48]. The similarity between the results obtained in our study and that of Babiloni and his coworkers [46] strongly support the hypothesis that the difference between conditions recorded from frontal and central scalp sites in our study was due to a cortical motor activation during observation of painting images. The negative ERP recorded during the observation of Paintings could represent the cortical motor representation of the hand gestures whose consequences, the brushstrokes, are directly observed by participants.

Most ERP studies of cortical motor functions use, as electrophysiological marker of motor activation, the lateralized readiness potential (LRP), an anticipatory potential preceding movement execution [49], [50]. The presence of this negative ERP component was also demonstrated during the observation of grasping actions [51], [52], [53]. However, a necessary condition for the occurrence of RPs is the expectancy of an upcoming movement, whether executed or observed, while our study did not include any explicit motor condition. As a consequence we cannot compare our study with those investigating the activation of the cortical motor system by means of RPs. Nevertheless, we can relate our results to other EEG studies investigating the activation of the sensory motor cortical circuits elicited by presentation of visual stimuli.

A recent EEG study based on microstates analyses [54] presented video-clips showing hand grasping with different grips and contexts, suggesting different intentions of the agent. The authors individuated a specific microstate the duration of which was modulated by the observed type of grip, in a time window between 200 and 300 ms after stimulus presentation. That time window is comparable to the one used in our study.

Cortical motor activation in response to observation of static handmade traces was found in a previous study [33] in which the frequency spectrum of MEG activity was analyzed comparing handwritten letters with printed letters presentation. Stronger ≈ 20 Hz suppression was recorded during the observation of handwritten letters than with that of printed letters These authors conclude that such cortical motor activation simulates the hand gestures of writing, thus allowing the observer to recognize handwritten scripts accurately. Our results cohere with these findings and, in addition, suggest a generalization of their conclusions: that the motor representation of the gesture implied in a perceived trace is part of the perceptual process even when the traces are devoid of symbolic significance.

The source localization analyses performed in our study identified many areas of activation, grouped into four different clusters, on the basis of their functional properties: visual, sensorimotor, prefrontal and orbitofrontal. Since the visual cluster lies beyond the aim of the present study, we will not discuss it here.

Our finding of a sensorimotor activation during observation of the visual traces of the movement that produced them in Kline’s paintings is in accordance with Gallese’s embodied simulation theory [27], [29]. Embodied simulation theory suggests that the activation of the same brain region both when executing actions and experiencing emotions and sensations and when witnessing others executing the same actions or experiencing the same emotions and sensations allows a pre-reflexive direct understanding of the actions, emotions and sensations of others. According to embodied simulation theory during art perception an empathic relationship is established between the beholder and the artwork [12], based on the activation of both mirror and canonical neurons. Some recent fMRI studies were in line with the hypothesis of a pivotal role of the embodied simulation in art perception. In particular, two recent fMRI experiments used as stimuli sculptures selected among masterpieces of Classical and Renaissance art [55], [56]. These studies showed the activation of the inferior parietal lobule and of the premotor cortex, and the authors interpreted these results as dependent on the intrinsic dynamic properties of the sculptures and on the sense of action that they evoked in the observer. We suggest that in our study the activation of the sensorimotor areas corresponded to an embodied simulation of the artist’s gesture triggered by dynamic components implicit in the visual appearance of the marks on the painting itself. The removal of these elements caused a significant reduction of sensorimotor activity. Furthermore, a recent behavioral study [57] demonstrated evidence for the activation of an embodied mechanism during the observation of abstract painting using a cognitive paradigm. The authors highlighted the role of action in abstract art perception showing that participants responded faster when their movement was compatible with the direction of the observed brushstrokes, even though the paintings were irrelevant to their task.

A recent EEG study [58] assessed the activation of the mirror mechanism during the observation of the modern non-figurative artwork by Lucio Fontana using the ERD (event-related sensory-motor alpha desyncronization) as index of motor simulation. The observation of Fontana artworks, when compared to the observation of the control images, was accompanied by a significant stronger ERD. Our study extends previous results and, in addition, localizes the cortical neural regions associated with the observation of the dynamic components of some examples of abstract art works. In principle, any visible traces of hand gestures could offer visual cues capable of triggering the beholder’s cortical sensory-motor representation of the same gesture − and not just abstract works of art. We hypothesize that what could make art different is perhaps, among other things, the artist’s ability to (mostly unconsciously) emphasize in the artwork those dynamic elements likely to activate beholders’ cortical motor representations of the very movements that produced those elements, like brushstrokes and color drops.

Kawabata and Zeki [3] already reported motor areas involvement during observation of paintings with different subjects (landscapes, portraits, still life and abstract paintings). Although their account of this activation did not refer to an involvement of the embodied mechanism, they suggested that perception of emotionally charged visual stimuli mobilizes the motor system, either to take some action to avoid the ugly or aversive stimulus or, in the case of beautiful stimuli, to make a response toward them.

Significantly, a recent neuropsychological study [59] found that lesions in the right hemisphere affected the “Assessment of Art Attributes” [60], that is, a neuropsychological instrument designed to assess six formal-perceptual and six conceptual-representational attributes of paintings in order to break down and quantify style and content of artworks. These authors reported that patients with lesions in the right BA 6 and BA 44 were characterized by a deviation in judgments of depth (one of the six formal-perceptual attributes), animacy and abstractness (two of the six conceptual-representational attributes). This finding, providing evidence of right motor areas contribution to art perception, is highly consistent with our results.

Finally, fMRI studies were performed with the aim to investigate the localization of brain areas activated by passive viewing of dance stimuli (videos) and to relate the activation of those areas with the aesthetic evaluation of the same stimuli [61], [62]. High esthetics ratings were correlated with increased activity, among others, in the right premotor cortex suggesting that right hemisphere sensorimotor areas might play a role in an esthetic response, not only to figurative art, but also to dance perception.

A third activated functional circuit was represented by the medial orbitofrontal and anterior cingulate cortices, corresponding to BA 11 and BA 32, brain regions related to reward processing [63]. The activation of these areas in response to art fits well with other studies [3], [9], [10], [55], [64] that interpreted the activity of such reward circuits as associated with the perception of pleasant and/or rewarding stimuli. An interesting study [65], performed a quantitative meta-analysis of 93 human neuroimaging studies of aesthetic processing across multiple sensory modalities and across both non-art and art objects. Brown and coworkers reached the conclusion that medial orbitofrontal cortex (BA 11) and the anterior cingulate cortex (BA 32) are cortical areas found to be active in relation with aesthetics processing.

The fourth group of cortical regions found to be active was represented by left prefrontal areas BA 8, BA 9, BA 10. These brain areas were also detected in other studies investigating responses to works of art [66], [7] and are commonly interpreted as being correlated with judgment tasks, especially when aesthetic parameters are involved [67]. Jacobsen and Hofel studied the time course of the general cognitive processes involved in aesthetic appreciation by means of EEG experiments [68], [69]. These authors presented a pool of abstract geometric patterns that varied in complexity and symmetry. On the basis of the occurrence of an early negative ERP, they established a temporal sequence for processes in a two-stage model. During the first stage, which takes place at around 300 ms after stimulus onset, an initial impression is formed. This process was associated with an anterior frontomedian activity, presumably associated with evaluative processes, and represents a fast impression formation. Interestingly, our results occurred in this early time window and this activation could correspond to a first level of aesthetic judgment. The second step was a deeper aesthetic evaluation beginning later in time, at close to 600 ms, and it was mainly related with an evaluative categorization process. In addition, a study [70] showed that the observation of works of art without stylistic information led to a greater activation mainly of a left prefrontal region (including areas BA 8, BA 6, BA 37, and BA 45), compared to the observation of artworks with stylistic information. These authors proposed two possible interpretations: one was that, when stylistic information was not available, participants may have had difficulties to categorize the stimuli and, therefore, tried to find related concepts within memory structures; another explanation could be a more verbally oriented processing of stimuli without provision of information and, therefore, a higher activation within the left hemisphere. Our task during the EEG session consisted of passive observation without any kind of explicit judgment of the images. The reason for this choice was related to our aim of assessing the involuntary and spontaneous activation of the cortical motor system in response to presentation of abstract paintings. Nevertheless, probably participants tended to make a first impression of the images that was more pronounced for the artworks, because they recognized them as such, as clearly demonstrated by the results of the “Artistic nature” questionnaire.

One of the main objectives of the present study was to detect the cortical neural regions activated by the perception of some abstract works of art. In particular we were mainly interested in detecting specific cortical areas activated by the observation of brushstrokes (clearly consequences of motor acts), compared to a similar graphic pattern deprived of these dynamic components. These components, visual traits like gradient and inhomogeneous signs, above defined as “dynamic”, altogether constitute the visual specificity of a brushstroke. Relevantly, our behavioral results indicated that the dynamic components embedded in the paintings were linked to participants’ subjective aesthetic experience. Indeed, a significant lower aesthetic score was given to the modified stimuli compared to paintings. At the same time, paintings were rated with a higher motion score, confirming their intrinsic dynamism. Our interpretation of the results is that the brushstrokes are responsible of the activation of sensorimotor areas controlling the motor acts that led to their production. Furthermore, source localization results showed that paintings observation was accompanied by motor and premotor areas activation.

We are mindful of a few caveats. First, the interpretation of the results is not univocal. In our view, sensorimotor involvement is directly associated with the presence of visible hand traces, while the activation of reward and categorizing associated areas is a consequence of the identification of the work of art as such.

Secondly, our study is focalized to a limited number of paintings by a single artist, which of course makes it difficult to draw conclusions about abstract art in general. Nevertheless, this experimental design hoped to set out a well-controlled and solid point of departure for further studies that would hopefully expand to a variety of stimuli representing more artists and styles.

## Conclusion

Our results show that the observation of abstract paintings by Franz Kline was accompanied by activation of premotor and motor areas, as well as by the activation of reward-related orbitofrontal areas, and cognitive categorization-related prefrontal areas. The sensorimotor activation elicited by observation of abstract paintings is an experimental evidence of the involvement of the cortical motor system in perception of static images belonging to abstract art. These results support the hypothesis postulating the role of embodied simulation of artist’s gestures in the perception of his/her works of art, grounded on the activation of the physiological mirror mechanism instantiated by cortical premotor areas. Orbitofrontal and left prefrontal activations in our view deal with the emotional and the cognitive dimensions of art perception, respectively.

## References

[1] Changeux JP (1994) Art and neuroscience. Leonardo 27: 189–201.

[2] Zeki S (1999) Inner Vision: An Exploration of Art and the Brain. New York: Oxford University Press. 224 p.

[3] Kawabata H, Zeki S (2004) Neural correlates of beauty. J Neurophysiol 91: 1699–1705. 15010496 10.1152/jn.00696.2003

[4] Cavanagh P (2005) The artist as neuroscientist. Nature 434: 301–307. 15772645 10.1038/434301a

[5] Conway BR, Livingstone MS (2007) Perspectives on science and art. Curr Opin Neurobiol 17: 476–482. 17851068 10.1016/j.conb.2007.07.010 PMC2813684

[6] Ramachandran VS, Hirstein W (1999) The science of art: A neurological theory of aesthetic experience. J. Conscious. Stud. 6: 15–51.

[7] Jacobsen T, Schubotz R, Hofel L, von Cramon D (2006) Brain correlates of aesthetic judgments of beauty. Neuroimage 29: 276–285. 16087351 10.1016/j.neuroimage.2005.07.010

[8] Cupchik GC, Vartanian O, Crawley A, Mikulis DJ (2009) Viewing artworks: contributions of cognitive control and perceptual facilitation to aesthetic experience. Brain Cogn 70: 84–91. 19223099 10.1016/j.bandc.2009.01.003

[9] Vartanian O, Goel V (2004) Neuroanatomical correlates of aesthetic preference for paintings. Neuroreport 9: 893–897. 10.1097/00001756-200404090-00032 15073538

[10] Lacey S, Hagtvedt H, Patrick VM, Anderson A, Stilla R, et al (2011) Art for reward’s sake: Visual art recruits the ventral striatum. Neuroimage 55: 420–433. 21111833 10.1016/j.neuroimage.2010.11.027 PMC3031763

[11] Freedberg D (1989) The Power of Images. Studies in the History and Theory of Response. Chicago: Chicago University Press. 534 p.

[12] Freedberg D, Gallese V (2007) Motion, emotion and empathy in esthetic experience. Trends Cogn Sci 11: 197–203. 17347026 10.1016/j.tics.2007.02.003

[13] Di Dio C, Gallese V (2009) Neuroaesthetics: a review. Curr Opin Neurobiol 19: 682–687. 19828312 10.1016/j.conb.2009.09.001

[14] Gallese V, Di Dio C (2012) Neuroesthetics: The Body in Esthetic Experience. In: Ramachandran V, editor. Encyclopedia of Human Behavior, 2^nd^ edition. San Diego: Elsevier Academic Press. pp. 687–693.

[15] Rizzolatti G, Fogassi L, Gallese V (2002) Motor and cognitive functions of the ventral premotor cortex. Curr Opin Neurobiol 12: 149–154. 12015230 10.1016/s0959-4388(02)00308-2

[16] Hurley SL (1998) Consciousness in action. London: Harvard University Press. 506 p.

[17] di Pellegrino G, Fadiga L, Fogassi L, Gallese V, Rizzolatti G (1992) Understanding motor events: a neurophysiological study. Exp Brain Res. 91: 176–180. 10.1007/BF00230027 1301372

[18] Gallese V, Fadiga L, Fogassi L, Rizzolatti G (1996) Action recognition in the premotor cortex. Brain 119: 593–609. 8800951 10.1093/brain/119.2.593

[19] Rizzolatti G, Fadiga L, Gallese V, Fogassi L (1996) Premotor cortex and the recognition of motor actions. Cogn Brain Res 3: 131–141. 10.1016/0926-6410(95)00038-0 8713554

[20] Gallese V, Keysers C, Rizzolatti G (2004) A unifying view of the basis of social cognition. Trends Cogn Sci 8: 396–403. 15350240 10.1016/j.tics.2004.07.002

[21] Fogassi L, Ferrari PF, Gesierich B, Rozzi S, Chersi F, et al (2005) Parietal lobe: from action organization to intention understanding. Science 29: 662–667. 10.1126/science.1106138 15860620

[22] Bonini L, Rozzi S, Serventi FU, Simone L, Ferrari PF, et al (2010) Ventral premotor and inferior parietal cortices make distinct contribution to action organization and intention understanding. Cereb Cortex 20: 1372–1385. 19805419 10.1093/cercor/bhp200

[23] Bonini L, Serventi FU, Simone L, Rozzi S, Ferrari PF, et al (2011) Grasping neurons of monkey parietal and premotor cortices encode action goals at distinct levels of abstraction during complex action sequences. J Neurosc 31: 5876–5886. 10.1523/JNEUROSCI.5186-10.2011 PMC6622840 21490229

[24] Rizzolatti G, Sinigaglia C (2010) The Functional Role of the Parieto-Frontal Mirror Circuit: Interpretations and Misinterpretations. Nat Rev Neurosci 11: 264–274. 20216547 10.1038/nrn2805

[25] Gallese V, Gernsbacher MA, Heyes C, Hickock G, Iacoboni M (2011) Mirror neuron Forum. Perspect Psychol Sci 6: 369–407. 25520744 10.1177/1745691611413392 PMC4266473

[26] de Vignemont F, Singer T (2006) The Emphatic Brain: How, When, and Why? Trends Cogn Sci 10: 435–441. 16949331 10.1016/j.tics.2006.08.008

[27] Gallese V (2003) The manifold nature of interpersonal relations: the quest for a common mechanism. Philos Trans R Soc Lond B Biol Sci 358: 517–528. 12689377 10.1098/rstb.2002.1234 PMC1693141

[28] Gallese V (2005) Embodied Simulation: From Neurons to Phenomenal Experience. Phenomenol Cogn Sci 4: 23–48.

[29] Gallese V, Sinigaglia C (2011) What is so special about embodied simulation? Trends Cogn Sci 15: 1–9. 10.1016/j.tics.2011.09.003 21983148

[30] Perry A, Bentin S (2009) Mirror activity in the human brain while observing hand movements: a comparison between EEG desynchronization in the mu-range and previous fMRI results. Brain Res 28: 126–132. 10.1016/j.brainres.2009.05.059 19500557

[31] Proverbio AM, Riva F, Zani A (2009) Observation of static pictures of dynamic actions enhances the activity of movement-related brain areas. PLoS One 4: 5389. 10.1371/journal.pone.0005389 PMC2671843 19421311

[32] Longcamp M, Anton JL, Roth M, Velay JL (2003) Visual presentation of single letters activates a premotor area involved in writing. Neuroimage 19: 1492–1500. 12948705 10.1016/s1053-8119(03)00088-0

[33] Longcamp M, Tanskanen T, Hari R (2006) The imprint of action: Motor cortex involvement in visual perception of handwritten letters. Neuroimage 33: 681–688. 16965922 10.1016/j.neuroimage.2006.06.042

[34] Longcamp M, Hlushchuk Y, Hari R (2011) What differs in visual recognition of handwritten vs. printed letters? An fMRI study. Hum Brain Mapp 32: 1250–1259. 20669164 10.1002/hbm.21105 PMC6870258

[35] Giromini L, Porcelli P, Viglione DJ, Parolin L, Pineda JA (2010) The feeling of movement: EEG evidence for mirroring activity during the observations of static, ambiguous stimuli in the Rorschach cards. Biol Psychol 85: 233–241. 20654683 10.1016/j.biopsycho.2010.07.008

[36] Pineda JA, Giromini L, Porcelli P, Parolin L, Viglione DJ (2011) Mu suppression and human movement responses to the Rorschach test. Neuroreport 22: 223–226. 21346645 10.1097/WNR.0b013e328344f45c

[37] Oldfield RC (1971) The assessment and analysis of handedness: the Edinburgh inventory. Neuropsychologia 9: 97–113. 5146491 10.1016/0028-3932(71)90067-4

[38] Yao D, Wang L, Arendt-Nielsen L, Chen AC (2007) The effect of reference choices on the spatio-temporal analysis of brain evoked potentials: the use of infinite reference. Comput Biol Med 37: 1529–1538. 17466967 10.1016/j.compbiomed.2007.02.002

[39] Pascual-Marqui RD (2002) Standardized Low-Resolution Brain Electromagnetic Tomography (sLORETA): Technical Details. Methods Find Exp Clin Pharmaco 24 Suppl. D: 5–12. 12575463

[40] Pascual-Marqui RD (2007) Discrete, 3D distributed, linear imaging methods of electric neuronal activity. Part 1: exact, zero error localization. arXiv:0710.3341 [math-ph],17. Available: http://arxiv.org/abs/0710.3341.

[41] Pascual-Marqui RD (2009) Theory of the EEG Inverse Problem. In: Tong S, Thakor NV, editors. Quantitative EEG Analysis: Methods and Clinical Applications. Boston: Artech House. Pp. 121–140.

[42] Fuchs M, Kastner J, Wagner M, Hawes S, Ebersole JS (2002) A standardized boundary element method volume conductor model. Clin Neurophysiol 113: 702–712. 11976050 10.1016/s1388-2457(02)00030-5

[43] Lancaster JL, Woldorff MG, Parsons LM, Liotti M, Freitas CS, et al (2000) Automated Talairach Atlas Labels For Functional Brain Mapping. Hum Brain Mapp 10: 120–131. 10912591 10.1002/1097-0193(200007)10:3<120::AID-HBM30>3.0.CO;2-8 PMC6871915

[44] Jurcak V, Tsuzuki D, Dan I (2007) 10/20, 10/10, and 10/5 systems revisited: their validity as relative head-surface-based positioning systems. Neuroimage 15: 1600–1611. 10.1016/j.neuroimage.2006.09.024 17207640

[45] Oostenveld R, Praamstra P (2001) The five percent electrode system for high-resolution EEG and ERP measurements. Clin Neurophysiol 112: 713–719. 11275545 10.1016/s1388-2457(00)00527-7

[46] Babiloni C, Del Percio C, Babiloni F, Carducci F, Cincotti F, et al (2003) Transient human cortical responses during the observation of simple finger movements: a high-resolution EEG study. Hum Brain Mapp 20: 148–157. 14601141 10.1002/hbm.10135 PMC6872072

[47] Babiloni C, Carducci F, Cincotti F, Rossini PM, Neuper C, et al (1999) Human movement-related potentials vs. desynchronization of EEG alpha rhythm: a high resolution EEG study. Neuroimage 10: 658–665. 10600411 10.1006/nimg.1999.0504

[48] Rossi S, Pasqualetti P, Rossini PM, Feige B, Ulivelli M, et al (2000) Effects of repetitive transcranial magnetic stimulation on movement-related cortical activity in humans. Cereb Cortex 10: 802–808. 10920051 10.1093/cercor/10.8.802

[49] Masaki H, Wild-Wall N, Sangals J, Sommer W (2004) The functional locus of the lateralized readiness potential. Psychophysiology 41: 220–230. 15032987 10.1111/j.1469-8986.2004.00150.x

[50] Bender S, Becker D, Oelkers-Ax R, Weisbrod M (2006) Cortical motor areas are activated early in a characteristic sequence during post-movement processing. Neuroimage 1: 333–351. 10.1016/j.neuroimage.2006.03.009 16698286

[51] Kilner JM, Vargas C, Duval S, Blakemore SJ, Sirigu A (2004) Motor activation prior to observation of a predicted movement. Nat Neurosci 7: 1299–1301. 15558063 10.1038/nn1355

[52] Fontana AP, Kilner JM, Rodrigues EC, Joffily M, Nighoghossian N, et al (2012) Role of the parietal cortex in predicting incoming actions. Neuroimage 59: 556–564. 21839178 10.1016/j.neuroimage.2011.07.046

[53] Goslin J, Dixon T, Fischer MH, Cangelosi A, Ellis R (2012) Electrophysiological examination of embodiment in vision and action. Psychol Sci 23: 152–157. 22241814 10.1177/0956797611429578

[54] Ortigue S, Sinigaglia C, Rizzolatti G, Grafton ST (2010) Understanding actions of others: the electrodynamics of the left and right hemispheres. A high-density EEG neuroimaging study. PLoS One 5: e12160. 20730095 10.1371/journal.pone.0012160 PMC2921336

[55] Di Dio C, Macaluso E, Rizzolatti G (2007) The golden beauty: brain response to classical and renaissance sculptures. PLoS One 11: e1201. 10.1371/journal.pone.0001201 PMC2065898 18030335

[56] Di Dio C, Canessa N, Cappa SF, Rizzolatti G (2011) Specificity of esthetic experience for artworks: an FMRI study. Front Hum Neurosci 5: 139. 22121344 10.3389/fnhum.2011.00139 PMC3220187

[57] Taylor JE, Witt JK, Grimaldi PJ (2012) Uncovering the connection between artist and audience: Viewing painted brushstrokes evokes corresponding action representations in the observer. Cognition 125: 26–36. 22986017 10.1016/j.cognition.2012.06.012

[58] Umiltà MA, Berchio C, Sestito M, Freedberg D, Gallese V (2012) Abstract art and cortical motor activation: an EEG study. Front. Hum. Neurosci 6: 311. 10.3389/fnhum.2012.00311 PMC3499799 23162456

[59] Bromberger B, Sternschein R, Widick P, Smith WII, Chatterjee A (2011) The right hemisphere in esthetic perception. Front Hum Neurosc 5: 109. 10.3389/fnhum.2011.00109 PMC3192953 22016728

[60] Chatterjee A, Widick P, Sternschein R, Smith WII, Bromberger B (2010) The assessment of art attributes. Emp Stud Art 28: 207–222.

[61] Calvo-Merino B, Jola C, Glaser DE, Haggard P (2008) Towards a sensorimotor aesthetics of performing art. Conscious Cogn 17: 911–922. 18207423 10.1016/j.concog.2007.11.003

[62] Bläsing B, Calvo-Merino B, Cross ES, Jola C, Honisch J, et al (2012) Neurocognitive control in dance perception and performance. Acta Psychol 139: 300–308. 10.1016/j.actpsy.2011.12.005 22305351

[63] Kringelbach ML (2005) The human orbitofrontal cortex: linking reward to hedonic experience. Nat Rev Neurosci 6: 691–702. 16136173 10.1038/nrn1747

[64] O’Doherty J, Winston J, Critchley H, Perrett D, Burt DM, et al (2003) Beauty in a smile: the role of medial orbitofrontal cortex in facial attractiveness. Neuropsychologia 41: 147–155. 12459213 10.1016/s0028-3932(02)00145-8

[65] Brown S, Gao X, Tisdelle L, Eickhoff SB, Liotti M (2011) Naturalizing aesthetics: brain areas for aesthetic appraisal across sensory modalities. Neuroimage 58: 250–258. 21699987 10.1016/j.neuroimage.2011.06.012 PMC8005853

[66] Cela-Conde CJ, Marty G, Maestu F, Ortiz T, Munar E, et al (2004) Activation of the prefrontal cortex in the human visual aesthetic perception. Proc Natl Acad Sci U S A 101: 6321–6325. 15079079 10.1073/pnas.0401427101 PMC395967

[67] Cela-Conde CJ, Agnati L, Huston JP, Mora F, Nadal M (2011) The neural foundations of aesthetic appreciation. Prog Neurobiol 94: 39–48. 21421021 10.1016/j.pneurobio.2011.03.003

[68] Jacobsen T, Höfel L (2003) Descriptive and evaluative judgment processes: behavioral and electrophysiological indices of processing symmetry and aesthetics. Cogn Affect Behav Neurosci 3: 289–299. 15040549 10.3758/cabn.3.4.289

[69] Höfel L, Jacobsen T (2007) Electrophysiological indices of processing aesthetics: Spontaneous or intentional processes? Int J Psychophysiol 65: 20–31. 17400317 10.1016/j.ijpsycho.2007.02.007

[70] Lengger PG, Fischmeister FP, Leder H, Bauer H (2007) Functional neuroanatomy of the perception of modern art: A DC-EEG study on the influence of stylistic information on aesthetic experience. Brain Res 1158: 93–102. 17559816 10.1016/j.brainres.2007.05.001

